# Quantitative Digitography Measures Motor Symptoms and Disease Progression in Parkinson’s Disease

**DOI:** 10.3233/JPD-223264

**Published:** 2022-09-02

**Authors:** Kevin B. Wilkins, Matthew N. Petrucci, Yasmine Kehnemouyi, Anca Velisar, Katie Han, Gerrit Orthlieb, Megan H. Trager, Johanna J. O’Day, Sudeep Aditham, Helen Bronte-Stewart

**Affiliations:** a Department of Neurology and Neurological Sciences, Stanford University School of Medicine, Stanford, CA, USA; b The Smith-Kettlewell Eye Research Institute, San Francisco, CA, USA; c Columbia University College of Physicians and Surgeons, New York City, NY, USA; d Department of Bioengineering, Stanford University, Stanford, CA, USA; e Department of Neurosurgery, Stanford University School of Medicine, Stanford, CA, USA

**Keywords:** Alternating finger tapping, cardinal motor signs, freezing of gait, keyboard, Parkinson’s disease, phenotype, remote measurement, rigidity, Unified Parkinson’s Disease Rating Scale, wearables

## Abstract

**Background:**

Assessment of motor signs in Parkinson’s disease (PD) requires an in-person examination. However, 50% of people with PD do not have access to a neurologist. Wearable sensors can provide remote measures of some motor signs but require continuous monitoring for several days. A major unmet need is reliable metrics of all cardinal motor signs, including rigidity, from a simple short active task that can be performed remotely or in the clinic.

**Objective:**

Investigate whether thirty seconds of repetitive alternating finger tapping (RAFT) on a portable quantitative digitography (QDG) device, which measures amplitude and timing, produces reliable metrics of all cardinal motor signs in PD.

**Methods:**

Ninety-six individuals with PD and forty-two healthy controls performed a thirty-second QDG-RAFT task and clinical motor assessment. Eighteen individuals were followed longitudinally with repeated assessments for an average of three years and up to six years.

**Results:**

QDG-RAFT metrics showed differences between PD and controls and provided correlated metrics for total motor disability (MDS-UPDRS III) and for rigidity, bradykinesia, tremor, gait impairment, and freezing of gait (FOG). Additionally, QDG-RAFT tracked disease progression over several years off therapy and showed differences between akinetic-rigid and tremor-dominant phenotypes, as well as people with and without FOG.

**Conclusions:**

QDG is a reliable technology, which could be used in the clinic or remotely. This could improve access to care, allow complex remote disease management based on data received in real time, and accurate monitoring of disease progression over time in PD. QDG-RAFT also provides the comprehensive motor metrics needed for therapeutic trials.

## INTRODUCTION

Parkinson’s disease (PD) is the fastest growing progressive neurological disorder in the world [1]. The treatment of PD requires complex, multi-dose medication regimens and constant monitoring due to adverse effects from medication or frequent visits for deep brain stimulation (DBS) programming. Descriptive data via patient messages or phone calls are often not adequate to make these complex management decisions. Instead, the assessment of a person with PD requires an in-person neurological examination to evaluate motor signs of rigidity (stiffness), bradykinesia (slowness), tremor, and gait and balance impairment. The need for an in-person assessment is most apparent for rigidity, which requires passive range of motion of the patient’s limbs by the examiner and cannot be done via video or with any wearable sensor.

The ideal solution for this problem would be a reliable remote objective measure that can be performed quickly, analyzed by an automated algorithm in real-time, and provide metrics for each of the cardinal motor signs of PD. Such a solution would improve access to care and provide comprehensive data to enable complex disease management decisions via telemedicine thus lessening the burden on neurologists. It would enable remote monitoring of disease progression over time, without requiring additional in person visits and could provide early diagnosis. Objective measures of rigidity in particular, would solve a critical unmet need for clinical trials that rely on video-based outcomes [2].

Although there have been major advances in the use of wearable devices for the monitoring of PD symptoms, none of them fully provide the vision described above. The Apple Watch-MM4PD was recently validated for remote monitoring of dyskinesia and tremor [3]. Similarly, the Parkinson’s KinetiGraph (PKG) also quantifies dyskinesias and tremor, as well as provides a measurement of bradykinesia [4, 5]. However, these wearable devices rely on the spectral analysis of movement which has not proven suitable for rigidity. Passive monitoring of typing on a computer keyboard has been taken as an alternative approach and shown promise to differentiate PD from healthy controls, but has not been validated against any of the cardinal motor signs in PD [6]. A key common problem across these wearable devices is that they require passive monitoring over longer periods of time from hours to days and often do not report symptoms in real-time.

The potential limitation of only passive monitoring via wearables has led to a growing effort to create short active tasks that can provide comprehensive assessment of PD motor symptoms. The Kinesia One uses a finger-worn device in conjunction with several movements to provide validated metrics of bradykinesia and tremor [7]. However, it does not provide an assessment of rigidity. Similarly, the growing prevalence of smartphones has led to creation of short tasks on phone-based apps. The most encouraging task to date for symptom assessment is variations of finger-tapping. Smartphone Tapper (SmT) and mPower (which includes several tasks in addition to finger-tapping including speech and cognitive evaluations) showed that finger-tapping provides metrics of total motor impairment, bradykinesia, and self-reported PD status [8, 9]. However, smartphone-based measures of tapping are limited to timing and position in space, but lack amplitude which is a key component of PD symptoms. Despite encouraging advances across wearable devices and smartphone-based apps, there is still no device or task that can provide reliable assessment of all the major cardinal motor signs in PD.

In this paper, we sought to evaluate whether the technology Quantitative DigitoGraphy (QDG) could provide objective measures of motor impairment in PD using metrics recorded during a repetitive alternating finger tapping (RAFT) task. We compiled data from several studies in which QDG-RAFT was used in a consistent off-therapy state and collected in a standardized manner. We found, in a large cohort of people with PD and healthy controls, that thirty seconds of QDG-RAFT reliably shows significant differences between these groups, provides correlated metrics for all the major cardinal motor signs of PD, including rigidity, bradykinesia, tremor, gait impairment, and freezing of gait (FOG), and tracks progression of such metrics over time. Additionally, we established that QDG-RAFT metrics show differences between akinetic rigid and tremor dominant PD phenotypes, and people with from those without FOG. QDG technology provides a unique and valuable companion to the suite of wearable sensors that are only validated for a subset of motor symptoms and require continuous data acquisition over the course of hours to days.

## MATERIALS AND METHODS

### Human subjects

Ninety-six individuals (52 males, 44 females) with clinically established PD [10] and forty-two healthy controls (20 males, 22 females) participated in the study. Data was collected in the Stanford Human Motor Control and Neuromodulation Laboratory. All experimental testing was done in the off-therapy state. Long- and short-acting medications were withdrawn over 24 to 48 and 12 h, respectively. If the patient had DBS, therapy was turned off for at least 15 min before testing began [11]. All participants gave written informed consent to participate in the study, which was approved by the Stanford University Institutional Review Board.

### Experimental protocol

Individuals performed repetitive alternating finger tapping (RAFT) on tensioned, engineered keys on a digitography device, which senses the amplitude displacement and timing of keystrikes. Individuals sat with their wrist resting on a pad at the same level as the keys of the device. They placed the index and middle finger on adjacent keys. The instructions were to press and release each key in an alternating pattern, as fast and regularly as possible for thirty seconds, starting and stopping only when they heard an auditory cue. Thirty seconds was chosen since sixty seconds was previously found to lead to fatigue in PD patients and controls, and ten seconds was found to be too short to demonstrate the progressive deterioration in performance, known as the sequence effect [12, 13]. They were instructed to attempt to press and release the keys completely. Participants performed the tasks with their eyes closed and while listening to white noise over headphones to remove visual and auditory feedback. Each subject had a short period of practice before the test began. Patients performed the task with each hand.

In addition to the RAFT task, individuals also performed the motor scale of the Unified Parkinson’s Disease Rating Scale (UPDRS III). Thirteen individuals performed the original UPDRS III and 83 individuals performed the Movement Disorders Society-UPDRS III (MDS-UPDRS III). Sub-scores for the UPDRS were defined as follows: 1) Bradykinesia: sum of finger tapping, hand movements, and pronation/supination items for the tested upper extremity, 2) Rigidity: rigidity item for the tested upper extremity, 3) Tremor: sum of postural and resting tremor items of the tested arm, 4) Gait & Freezing of Gait (FOG): sum of gait and freezing of gait items. An individual was considered a freezer if they scored >0 on either the Freezing of Gait Questionnaire (FOGQ) or exhibited freezing during gait assessments in the lab.

A subset of this cohort (N = 18) who were implanted with an investigative neurostimulator (Activatrademark PC+S, Medtronic, PLC) was followed longitudinally as part of a ‘washout’ study to assess symptom progression over time [14]. This involved testing in the off-therapy state, which entailed stopping long-acting dopamine agonists at least 48 h, dopamine agonists and controlled release carbidopa/levodopa at least 24 h, and short acting medication at least 12 h before testing, as well as the turning off of the DBS stimulator for at least 15 min. These individuals performed multiple RAFT experiments to assess changes in each of the QDG metrics over time. Tests were separated by a minimum of 3 months.

### Kinematic data acquisition and analysis

**Fig. 1:**
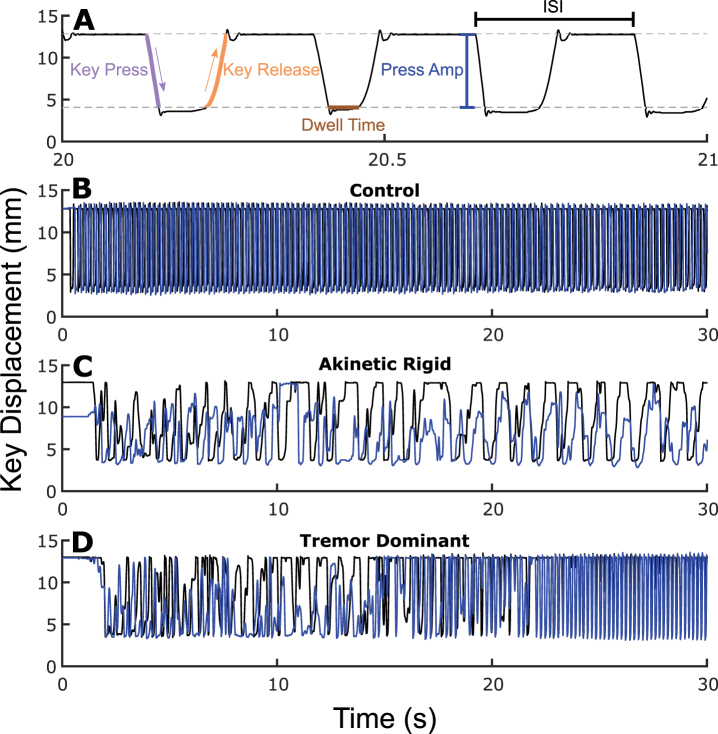
.(A) Zoomed in QDG trace. Gray dashed lines represent nonlinear zones. Examples of (B) healthy control, (C) akinetic rigid PD phenotype, and (D) tremor dominant PD phenotype with one finger in blue and the other in black. See online edition for color version.

The device produced a voltage signal that was proportional to the displacement of the key using an optic encoder [15]. The key displacement was linearly related to the output voltage signal with a resolution of 62.5μm per 40 mV. For key displacements less than 9 mm, the device operated in a linear zone. Near the base of the key displacement, the key reached a compliant mechanical stop where displacement was non-linearly related to the output voltage signal, which we defined as the “nonlinear zone”. A customized detection algorithm was used to determine specific states in the cycle of finger movement while ignoring the nonlinear zone (Fig. 1A). Six metrics were then calculated from these cycles: 1) Press amplitude [15, 16], 2) Press amplitude coefficient of variation (CV: standard deviation/mean) [15, 16], 3) Inter-strike interval (ISI: time to complete one cycle of finger movement) [12, 15–17], 4) Inter-strike interval CV (ISI CV) [12, 15–17], 5) Release slope (i.e., ratio of the amplitude of the key release compared to duration of release) [18], and 6) Dwell time (i.e., duration at bottom of the press) [17]. Release amplitude and release amplitude CV were not investigated due to high collinearity with press amplitude (*R* > 0.99) and press amplitude CV (*R* > 0.99). The average for each of these metrics across the thirty second trial was computed for each finger and then averaged across fingers for each hand. Each hand was treated separately.

### Statistical analysis

Statistical analyses were run in R (version 3.6.0, R Foundation for Statistical Computing, University of Auckland, New Zealand) and MATLAB (version 9.9, Mathworks, Natick, MA, USA). To assess differences in the six metrics between individuals with PD and healthy controls, a linear mixed effects model was used with fixed effects of group (PD vs. C) and age, as well as a random intercept for subject. Sex was not included as a fixed effect as it did not have a significant effect for any of the 6 metrics in the healthy control data. A Pearson correlation was computed between total MDS-UPDRS III and each of the 6 metrics. Spearman correlations were used to assess correlations between sub-scores of the UPDRS and the 6 metrics. To assess changes over time, a linear mixed effects model was used with fixed effects of time and age, a random slope of time, and random intercept for subject. To measure differences in phenotypes (i.e., akinetic rigid [AR] vs. tremor dominant [TD]), a linear mixed effects model was performed on the longitudinal cohort with fixed effects of phenotype and age, along with a random slope of time and random intercept for subject for each of the 6 metrics. Lastly, a similar linear mixed effects model but with fixed effects of subtype (freezer vs. non-freezer) and age was calculated to evaluate differences for each of the 6 metrics between freezers and non-freezers. Significance was set at *p* < 0.0083 (.05/6) due to a Bonferroni correction for the six metrics tested.

## RESULTS

### Demographic characteristics

Demographic information for the individuals with PD and Controls is shown in Table 1. The PD group had a mean MDS-UPDRS III (N = 83) of 25.1±13.4 and disease duration of 8.7±5.7 years. The healthy controls were younger (60.0±9.0 years) on average than the participants with PD (65.1±9.1 years), but age was controlled for when evaluating group differences in metrics.

**Table 1:** Participant demographics

	Controls	PD
*N*	42	96
Age	60.0±9.0 [44.0–76.0]	65.1±9.1 [34.4–90]
Sex	20 M, 22 F	52 M, 44 F
MDS-UPDRS III	–	25.1±13.4 [4–71]
UPDRS III	–	31.6±9.7 [12–49]
Disease duration	–	8.7±5.7 [1–22]

### Finger tapping metrics differ between individuals with PD and healthy controls

Figure 1 depicts individual QDG-RAFT traces from a healthy control (Fig. 1B) and two individuals with PD, one with the akinetic rigid (AR) phenotype (Fig. 1C) and one with the tremor dominant (TD) phenotype (Fig. 1D).

The alternating finger tapping in the healthy control is characterized by rhythmic, rapid, full amplitude keystrikes for the duration of the thirty seconds. The AR individual displays small and variable amplitudes of presses and releases throughout the trace, slower overall tapping speed (longer ISIs), and a slower release slope of the key. However, they maintain an alternating finger tapping pattern. The TD individual, Fig. 1D, also initially displays alternating finger tapping, with variability in tapping amplitude. After ∼20 s, involuntary tremor over-rode the voluntary tapping as evident by the high frequency (>4 Hz), full amplitude, short duration, non-alternating strikes.

**Fig. 2:**
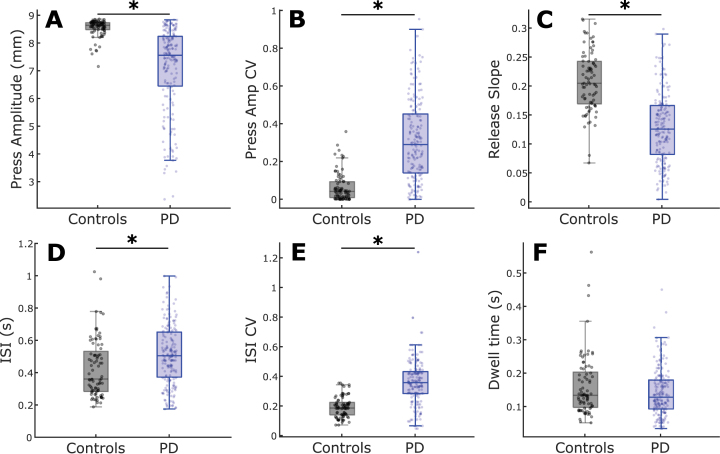
Comparison of QDG metrics between PD and controls. Boxplots with individual data overlaid for (A) Press amplitude, (B) Press amplitude CV, (C) Release slope, (D) ISI, (E) ISI CV, and (F) Dwell time. ^*^indicates significant differences between groups. See online edition for color version.

Group differences between PD and controls for each of the six metrics were explored, while controlling for age (Fig. 2). Press amplitude (*t* = 6.60, *p* = 2.70e-10), press amplitude CV (*t* = 7.96, *p* = 7.54e-14), ISI CV (*t* = 7.94, *p* = 9.23e-14), release slope (*t* = 7.20, *p* = 8.29e-12), and ISI (*t* = 2.83, *p* = 0.0050) showed significant differences between PD and controls (Fig. 1A-E). There was no difference in Dwell time (*t* = 1.49, *p* = 0.14) between groups (Fig. 1F). An increase in the CV in a metric signifies greater variability.

### QDG-RAFT metrics correlate with cardinal motor signs and gait impairment

Individuals who performed the original UPDRS (N = 26) were not included in the correlations with the total MDS-UPDRS III or gait impairment and FOG scores. There were significant associations between total MDS-UPDRS III (N = 166) and press amplitude (R = –0.38, *p* = 9.51e-7), ISI CV (R = 0.36, *p* = 5.84e-6), press amplitude CV (R = 0.26, *p* = 0.0012), and release slope (R = –0.23, *p* = 0.0048). Dwell time and ISI did not show a significant association (Table 2).

**Table 2:** Correlation results

UPDRS score	QDG metric	Correlation coef	*p*
Total	**Press amplitude**	**–0.38**	**9.51e-7**
	**ISI CV**	**0.36**	**5.84e-6**
	**Press amplitude CV**	**0.26**	**0.0012**
	**Release slope**	**–0.23**	**0.0048**
	ISI	0.19	0.010
	Dwell time	0.19	0.021
Rigidity sub-score	**Release slope**	**–0.43**	**1.63e-9**
	**Dwell time**	**0.27**	**1.88e-4**
	**Press amplitude**	**–0.25**	**7.04e-4**
	**ISI**	**0.21**	**0.0035**
	**Press amplitude CV**	**0.21**	**0.0049**
	ISI CV	–0.070	0.35
Bradykinesia sub-score	**Press amplitude**	**–0.34**	**2.60e-6**
	**Release slope**	**–0.33**	**4.80e-6**
	**Press amplitude CV**	**0.28**	**1.18e-4**
	ISI CV	0.15	0.051
	Dwell time	0.12	0.12
	ISI	–0.018	0.81
Tremor sub-score	**Dwell time**	**–0.26**	**4.90e-4**
	ISI	–0.13	0.072
	Release slope	0.13	0.079
	ISI CV	0.10	0.18
	Press amplitude CV	0.10	0.18
	Press amplitude	–0.069	0.36
Gait & FOG sub-score	**ISI CV**	**0.28**	**4.77e-4**
	**Release slope**	**–0.26**	**0.0013**
	Press amplitude	0.21	0.0084
	Press amplitude CV	0.16	0.052
	Dwell time	0.14	0.074
	ISI	0.055	0.48

To determine if there was any relationship between QDG-RAFT metrics and specific cardinal motor signs, we quantified correlations between rigidity, bradykinesia, and tremor sub-scores across both hands of the full cohort (N = 192) (Table 2). There were significant associations between the rigidity sub-score and release slope (*ρ*= –0.43, *p* = 1.63e-9), Dwell time (*ρ*= 0.27, *p* = 1.88e-4), press amplitude (*ρ*= –0.25, *p* = 7.04e-4), ISI (*ρ*= 0.21, *p* = 0.0035), and press amplitude CV (*ρ*= 0.21, *p* = 0.0049). The bradykinesia sub-score significantly correlated with press amplitude (*ρ*= –0.34, *p* = 2.60e-6), release slope (*ρ*= –0.33, *p* = 4.80e-6), and press amplitude CV (*ρ*= 0.28, *p* = 1.18e-4). Tremor sub-score was significantly associated with Dwell time (*ρ*= –0.26, *p* = 4.90e-4). Gait impairment and FOG sub-scores (MDS-UPDRS III cohort only) significantly correlated with ISI CV (*ρ*= 0.28, *p* = 4.77e-4) and release slope (*ρ*= –0.26, *p* = 0.0013).

### QDG-RAFT performance deteriorates over time

**Fig. 3:**
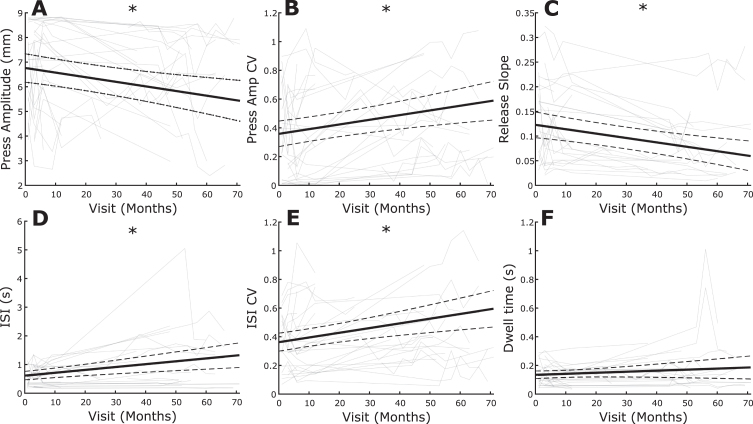
Average change over time (thick black line) with individual data overlaid (light gray) for (A) Press amplitude, (B) Press amplitude CV, (C) Release slope, (D) ISI, (E) ISI CV, and (F) Dwell time. ^*^indicates significance. Dashed lines represent 95% confidence interval.

Eighteen individuals were followed longitudinally to assess changes in QDG metrics over time, Fig. 3. This group was followed for an average of 35.5±27.0 months, and up to six years, with an average number of visits over that time of 4.6±2.7 (Table 3). Variability in length of participation in this longitudinal assessment was primarily from lack of re-implantation of the investigative neurostimulator, late enrollment in the study, or prioritization of other experimental tasks as part of a larger investigative neurostimulator study. Press amplitude (*t* = 4.02, *p* = 8.94e-5), press amplitude CV (*t* = 3.91, *p* = 1.38e-4), release slope (*t* = 4.77, *p* = 4.17e-6), ISI (*t* = 3.96, *p* = 1.14e-4), and ISI CV (*t* = 3.78, *p* = 2.26e-4) all significantly worsened over time (Fig. 3). Meanwhile, Dwell time (*t* = 1.78, *p* = 0.077) did not significantly change.

**Table 3:** Longitudinal participant demographics

Patient	Age	Sex	Phenotype	Subtype	# Sessions	Total months
1	73	M	TD	C	4	12
2	53	M	AR	NF	8	63
3	63	M	TD	C	10	69
4	66	M	TD	NF	5	52
5	58	M	AR	F	8	66
6	42	M	TD	NF	3	6
7	69	M	TD	F	1	1
8	54	M	AR	F	3	48
9	72	M	TD	C	4	51
10	59	F	AR	F	3	37
11	34	M	AR	F	1	1
12	57	M	AR	F	1	1
13	68	F	AR	NF	5	71
14	62	M	TD	NF	2	6
15	55	F	AR	NF	7	55
16	57	F	TD	F	8	63
17	56	M	AR	F	4	12
18	51	M	AR	F	6	25
Avg±SD	58.3±10.0				4.6±2.7	35.5±27.0

AR, akinetic rigid; C, converter (non-freezer to freezer); F, freezer; NF, non-freezer; TD, tremor dominant.

### Differences in QDG-RAFT metrics between AR and TD PD phenotypes

**Fig. 4:**
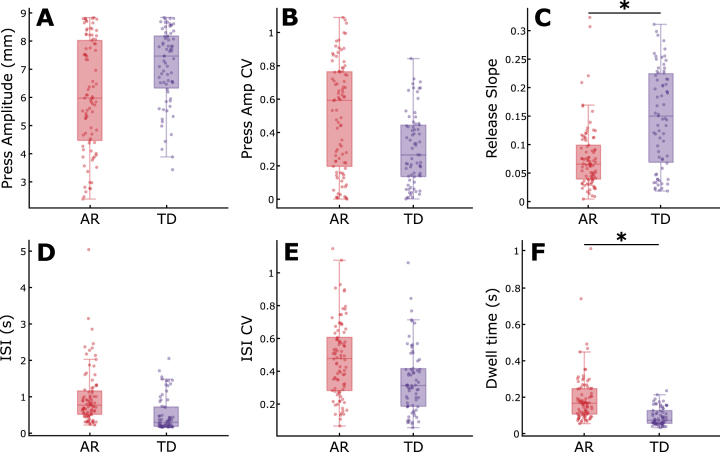
Comparison of QDG metrics between akinetic rigid and tremor dominant phenotypes. Boxplots with individual data overlaid for (A) Press amplitude, (B) Press amplitude CV, (C) Release slope, (D) ISI, (E) ISI CV, and (F) Dwell time. ^*^indicates significant differences between groups. See online edition for color version.

Within the longitudinal cohort, there was a significant difference between AR and TD phenotypes for release slope (*t* = 2.74, *p* = 0.0068) and Dwell time (*t* = 3.41, *p* = 8.37e-4) when controlling for age (Fig. 4). The AR phenotype demonstrated slower release slopes and longer keystrike durations that the TD phenotypes. There were no differences between phenotypes for press amplitude, press amplitude CV, ISI, or ISI CV. These results remained consistent when controlling for total motor impairment by including total MDS-UPDRS III as an additional fixed effect in the mixed model in addition to age (see Supplementary Table 1).

### Freezers demonstrate worse finger tapping performance compared to non-freezers

**Fig. 5:**
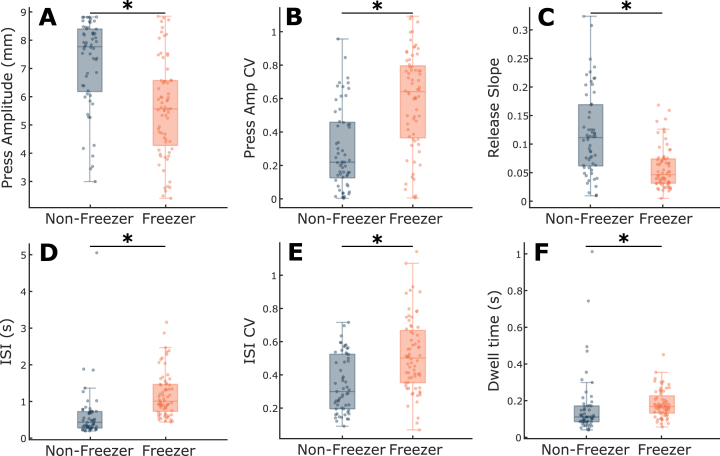
Comparison of QDG metrics between freezer and non-freezer subtypes. Boxplots with individual data overlaid for (A) Press amplitude, (B) Press amplitude CV, (C) Release slope, (D) ISI, (E) ISI CV, and (F) Dwell time. ^*^indicates significant differences between groups. See online edition for color version.

A subset of the longitudinal cohort (N = 15) was used to assess differences between individuals with FOG (freezers) and those without evidence of FOG (non-freezers) (Fig. 5). Individuals who converted from a non-freezer to a freezer during the period (N = 3) were excluded from the analysis. Freezers were significantly worse across all six QDG metrics, including press amplitude (*t* = 3.22, *p* = 0.0017), press amplitude CV (*t* = 3.47, *p* = 7.29e-4), release slope (*t* = 4.32, *p* = 3.17e-5), ISI (*t* = 3.89, *p* = 1.63e-4), ISI CV (*t* = 2.90, *p* = 0.0044), and Dwell time (*t* = 4.55, *p* = 1.30e-5) when controlling for age. These results remained consistent when controlling for total motor impairment by including total MDS-UPDRS III as an additional fixed effect in the mixed model in addition to age (see Supplementary Table 2).

## DISCUSSION

The current large study has demonstrated that a thirty second repetitive alternating finger tapping (RAFT) task on an engineered digitography device that accurately measures amplitude and timing reliably showed substantial differences between people with PD and healthy controls. Quantitative digitography (QDG) produced metrics that were correlated with overall motor disability (total MDS-UPDRS III score), and with all the major cardinal motor signs of PD, including rigidity, bradykinesia, tremor, gait impairment and FOG. QDG technology reliably tracked motor progression over several years, off therapy. Furthermore, QDG-RAFT metrics showed differences between akinetic rigid and tremor dominant phenotypes, as well as between freezers and non-freezers. Together, these results support the potential viability of QDG-RAFT as a tool for providing quantitative measurement of PD motor signs. The task is simple, takes thirty seconds, and does not require continuous monitoring like many wearables. QDG technology and the rigidity metric provide a critically important supplement to video MDS-UPDRS assessments of PD in clinical trials.

### Differences between PD and healthy controls

Individuals with PD had significantly smaller and more variable press amplitudes, slower release amplitude slopes, and slower and more arrhythmic tapping than age matched healthy controls. These findings build on prior work showing the effectiveness of an alternating finger tapping task to differentiate individuals with PD from controls [8, 12, 17, 18]. Our lab’s original RAFT findings were found on a MIDI keyboard, which only primarily registered full amplitude strikes [12, 17]. The fact that we were able to replicate these findings and build on them with a newly developed digitography device that also tracts amplitude displacement further supports the utility of RAFT as an assessment of PD symptoms. The average disease duration of the cohort studied was 8.7±5.7 years, which reflects moderate to advanced stage PD. We have previously demonstrated that QDG metrics were different from healthy controls in very early stage PD, within 1 year of diagnosis and before any symptomatic therapy had been started [19]. We also demonstrated a trend for QDG-RAFT to show progression of disease when performed remotely once a week for six months in untreated very early stage PD [20]. In addition, RAFT was shown to be the most effective motor predictor for conversion from idiopathic REM sleep behavior disorder to PD, with deficits observed nine years prior to conversion [21].

The effectiveness of differentiating PD from controls has led to the widespread implementation of finger tapping on smart phone battery of assessments for PD [3, 8, 9, 22, 23]. However, smartphone applications are limited to temporal metrics of tapping and position in 2D space of the fingers [9]. Our current results demonstrate that amplitude-based metrics are critical components for differentiating aspects of Parkinsonism motor deficits, especially rigidity and bradykinesia.

### QDG-RAFT correlates of MDS-UPDRS III and its sub-scores

One of the major results from this study is that the 30-s QDG-RAFT was able to generate metrics that significantly correlated with overall motor impairment in PD, as well as with the major cardinal signs of PD: rigidity, bradykinesia, tremor, gait impairment, and FOG. Our lab recently demonstrated that the QDG-RAFT release slope significantly correlated with rigidity of the upper extremity in a smaller cohort, which we replicated here [18]. One possible mechanism is that the release of the key is partly a passive movement: the tensioned key partially pushes the finger up as the key is restored to its neutral position. Increased rigidity or stiffness will lead to slower releases and lower release slopes. The ability to quantitatively assess rigidity is a major advancement since rigidity cannot be assessed remotely via video. Successful efforts to develop remote devices for assessing rigidity have been limited and none can also measure the other cardinal PD motor signs [24–28]. Typical wearable inertial monitoring units (IMUs) or smart watches use spectral and kinematic analyses of movement and are unable to measure rigidity [3, 4].

Lower press amplitude and variability in both the amplitude and temporal domains were critical metrics related to the MDS-UPDRS bradykinesia score, which represents the well-known hypometria and sequence effect in clinical assessments of repetitive finger tapping and other movements in PD. With QDG these can now be assessed independently unlike in the MDS-UPDRS III. Keystrike amplitude and amplitude variability have also distinguished moderate PD and people with chronic HIV from controls [16].

Higher tremor scores were related to shorter Dwell times during RAFT. This is a result of the involuntary nature of tremor, which when manifest on the keys, produces rhythmic involuntary high frequency tapping with a very short period of time between the press and release phases (Dwell time).

QDG-RAFT arrhythmicity (ISI CV) and release slope were significantly correlated with the MDS-UPDRS III items of gait impairment and FOG. Arrhythmicity of gait is a fundamental aspect of gait impairment and a predictor of FOG, falls and related postural deficits [29, 30]. This demonstrates the system-wide nature of aberrant motor control in PD in which arrhythmicity and freezing of gait may manifest themselves as arrhythmicity in finger tapping.

### QDG-RAFT documents the progression of PD motor disability over several years

To our knowledge, this is the first study of its kind to track quantitative metrics related to the major cardinal signs of PD longitudinally for several years. We found that individuals got slower, more irregular, stiffer, and moved with smaller amplitude, as would be expected with progressive parkinsonism in the off-therapy state. Although longitudinal quantitative tracking has been successfully applied to aspects of gait [31–33], assessments of overall motor impairment and symptoms have been mainly limited to clinical assessments that have low reliability [34]. The MM4PD smart watch system was used to track symptoms over time, but that was limited to six months and only examined tremor and dyskinesias on therapy [3]. Analysis of the hold and release times of daily computer keyboard use in people with PD was evaluated to document treatment response over time, but the keyboard metric was not related to any PD motor sign and the duration of the longitudinal study was only six months [35]. To date, most studies investigating disease progression over time have used the MDS-UPDRS III [36]. While this is a comprehensive clinical assessment, the substantial intra- and inter-rater variability and the requirement for an in-person evaluation demonstrates the need for an additional rapid, remote, and reliable technology that tracks progression of all cardinal motor signs [34]. Incorporating QDG technology into clinical practice and clinical trials marks a valuable step forward in being able to both understand progression of different Parkinsonian symptoms over time and create data-informed personalized treatment plans for optimizing therapy.

### QDG-RAFT metrics show differences between phenotypes and freezer subtypes

QDG-RAFT metrics showed differences between akinetic rigid (AR) and tremor dominant (TD) PD phenotypes. Specifically, the AR cohort had slower release slopes, indicative of greater rigidity, and the TD cohort had shorter Dwell time, suggesting greater number of short duration strikes. These findings matched the initial correlations between QDG metrics and UPDRS sub-scores. We originally identified short durations of keystrikes on a MIDI keyboard, along with tapping frequencies of 4+ Hz as signatures of tremor [12]. Interestingly lower release slopes were also correlated with gait impairment and FOG and AR phenotypes may be more likely to exhibit gait deficits [37, 38].

PD participants identified as freezers from gait tasks and the FOGQ were significantly worse in their performance of QDG-RAFT amplitude, release slope, frequency, and regularity metrics than non-freezers, even when controlling for overall motor impairment. The topic of whether freezing of upper limb (FO-UL) movement is related to FOG has been debated [39–43]. Arrhythmicity of gait (stride time CV) is a robust correlate of FOG [44–46]. A study of bimanual repetitive drawing did demonstrate episodes of FO-UL correlated with FOG, but without major arrhythmicity [39]. We believe a complex unimanual task may be more effective for evoking freezing behavior since we previously found that unimanual QDG, but not bimanual single finger tapping, elicited arrhythmicity [12, 15]. The significance of all six QDG-RAFT metrics likely reflects the different aspects of freezing: e.g., cessation of movement (reflected in longer ISI and smaller amplitude of presses) and arrhythmicity (reflected in higher press amplitude CV and ISI CV).

### Limitations

The testing here was limited to off-therapy and therefore dyskinesias were not present during any of the testing. Future on-therapy studies could assess whether it is possible to assess dyskinesia in the tested limb with QDG metrics. There was an age difference between groups, but age was controlled for in the statistical comparisons between groups. Due to the difficulty in acquiring longitudinal washout data (i.e., withdrawal of medication and deep brain stimulation), the longitudinal follow-up assessments were only done on a subset of the overall cohort as part of a separate study. We have demonstrated reliability of QDG-RAFT performed weekly [20] and further studies will need to be done to assess the test-retest validity of QDG on shorter time-scales.

### Conclusion

A simple, short QDG-RAFT task provided quantitative metrics which correlated with all the cardinal motor signs of PD, including rigidity and gait impairment/FOG, tracked disease progression over time, and showed differences between PD phenotypes. These findings highlight the potential for QDG-RAFT as an objective measurement tool that could be used either in clinic or remotely to provide tracking of all of the PD cardinal motor signs, only some of which are currently available from passively sensing wearable devices.

## Supplementary Material

Supplementary Material
