# Supplementary material for: Quantitative Digitography Measures Motor Symptoms and Disease Progression in Parkinson’s Disease
Source: J Parkinsons Dis. 2022 Sep 2;12(6):1979–90. doi: 10.3233/JPD-223264 (PMC9535590; doi:10.3233/JPD-223264)
Supplement: Supplementary Material [file jpd-12-jpd223264-s001.pdf]

Supplementary Material

Quantitative Digitography Measures Motor Symptoms and Disease Progression in Parkinson's Disease

Supplementary Table 1. Phenotype Differences Corrected for Impairment Level

QDG Metric	T-value	p
**Dwell Time**	**4.17**	**4.94e-5**
**Release Speed**	**2.80**	**0.0057**
Press Amplitude CV	2.08	0.039
ISI	1.94	0.054
Press Amplitude	1.86	0.065
ISI CV	1.32	0.10

Supplementary Table 2. Freezer Differences Corrected for Impairment Level

QDG Metric	T-value	p
**Release Speed**	**4.34**	**3.00e-5**
**Dwell Time**	**3.87**	**1.47e-4**
**Press Amplitude CV**	**3.08**	**0.0025**
**ISI**	**2.96**	**0.0038**
**ISI CV**	**2.91**	**0.0043**
**Press Amplitude**	**2.69**	**0.0081**
